# Comparative genome sequencing and analyses of *Mycobacterium cosmeticum* reveal potential for biodesulfization of gasoline

**DOI:** 10.1371/journal.pone.0214663

**Published:** 2019-04-09

**Authors:** Wei Yee Wee, Avirup Dutta, Jayasyaliny Jayaraj, Siew Woh Choo

**Affiliations:** 1 Monash University Malaysia, School of Science, Bandar Sunway, Malaysia; 2 The Novo Nordisk Foundation Center for Basic Metabolic Research, Human Genomics and Metagenomics in Metabolism, Faculty of Health and Medical Sciences, University of Copenhagen, Copenhagen, Denmark; 3 Department of Biological Sciences, Xi’an Jiaotong-Liverpool University, Suzhou Dushu Lake Science and Education Innovation District, Suzhou Industrial Park, Suzhou, P. R. China; 4 Suzhou Genome Centre (SGC), Health Technologies University Research Centre (HT-URC), Xi’an Jiaotong-Liverpool University, Suzhou Dushu Lake Science and Education Innovation District, Suzhou Industrial Park, Suzhou, P. R. China; Jamia Hamdard, INDIA

## Abstract

*Mycobacterium cosmeticum* is a nontuberculous *Mycobacterium* recovered from different water sources including household potable water and water collected at nail salon. Individual cases of this bacterium have been reported to be associated with gastrointestinal tract infections. Here we present the first whole-genome study and comparative analysis of two new clinically-derived *Mycobacterium* sp. UM_RHS (referred as UM_RHS after this) and *Mycobacterium* sp. UM_NYF (referred as UM_NYF after this) isolated from patients in Indonesia and Malaysia respectively to have a better understanding of the biological characteristic of these isolates. Both strains are likely *Mycobacterium cosmeticum* as supported by the evidence from molecular phylogenetic, comparative genomic and Average Nucleotide Identity (ANI) analyses. We found the presence of a considerably large number of putative virulence genes in the genomes of UM_RHS and UM_NYF. Interestingly, we also found a horizontally transferred genomic island carrying a putative *dsz* operon proposing that they may have potential to perform biodesulfization of dibenzothiophene (DBT) that may be effective in cost reduction and air pollution during fuel combustion. This comparative study may provide new insights into *M*. *cosmeticum* and serve as an important reference for future functional studies of this bacterial species.

## Introduction

*Mycobacterium* is a genus under the actinobacteria phylum classified together with other well-known human pathogens like *M*. *tuberculosis* (causing tuberculosis) and *M*. *leprae* (causing leprosy) [1–3]. This genus consists of another group of mycobacteria known as the nontuberculous mycobacteria (NTM). NTM has been associated with human diseases and was first reported in pathological human secretions in 1884 [4]. It represents a diverse group of environmentally opportunistic human pathogens widely found at peat-rich potting soil, drinking water in buildings and households, on animals and also in food [5–7]. The NTM can cause human infections mainly occurring under environmental exposures, sternal wound infections, plastic surgery wound infections, or post-injection abscesses [8–10].

*M*. *cosmeticum* is usually recovered from water including household potable water [11] and water collected at the nail salon [12] and activated sludge from wastewater treatment [13]. A case where *M*. *cosmeticum* has been implicated as a gastrointestinal tract pathogen causing ascites in a 63-year-old woman [14] has been reported. Moreover, it has also been reported that this bacterium has induced severe diffuse granulomatous colitis in a non-immunocompromised 32-year-old Turkish patient [15].

In this paper, we sequenced two new clinically-derived *Mycobacterium* sp. UM_RHS (referred as UM_RHS after this) and *Mycobacterium* sp. UM_NYF (referred as UM_NYF after this). We have also performed bioinformatics analyses particularly, comparative analyses to further understand the genomics, phylogeny and biology of this bacterial species. The genome sequences of UM_RHS and UM_NYF have been deposited at GenBank with the accession numbers of GCA_000455185.1 and GCA_000987455.1, respectively.

## Results and discussion

### Genome sequencing and assembly

The genome of UM_RHS sequenced using Illumina HiSeq 2000 sequencing technology yielded 51,391,676 paired-end (PE) reads. 50,813,644 usable reads were obtained after quality based trimming with Phred score of 20 and removal of exact duplicates and reverse complement duplicate reads using PRINSEQ lite version 0.20 [16]. The *de novo* assembly of these reads generated 167 contigs with a total genomic length of 6,775,899bp and G+C content of 67.9%. This UM_RHS assembly has a N50 value of 95,298bp with minimum contig length of 510bp and maximum contig size of 242,034bp, suggesting considerably high quality of this assembly for downstream analyses.

Similar to the UM_RHS, the genome of UM_NYF sequenced with the same sequencing platform yielded 39,868,088 raw PE reads. After the filtering steps, 39,529,963 preprocessed reads were used for assembly. The assembly of UM_NYF genome resulted in 332 contigs with a total genomic length of 6,809,253bp and a G+C content of 67.9% and N50 value of 61,947bp.

### Recognition of Isolated Species

**Fig 1:**
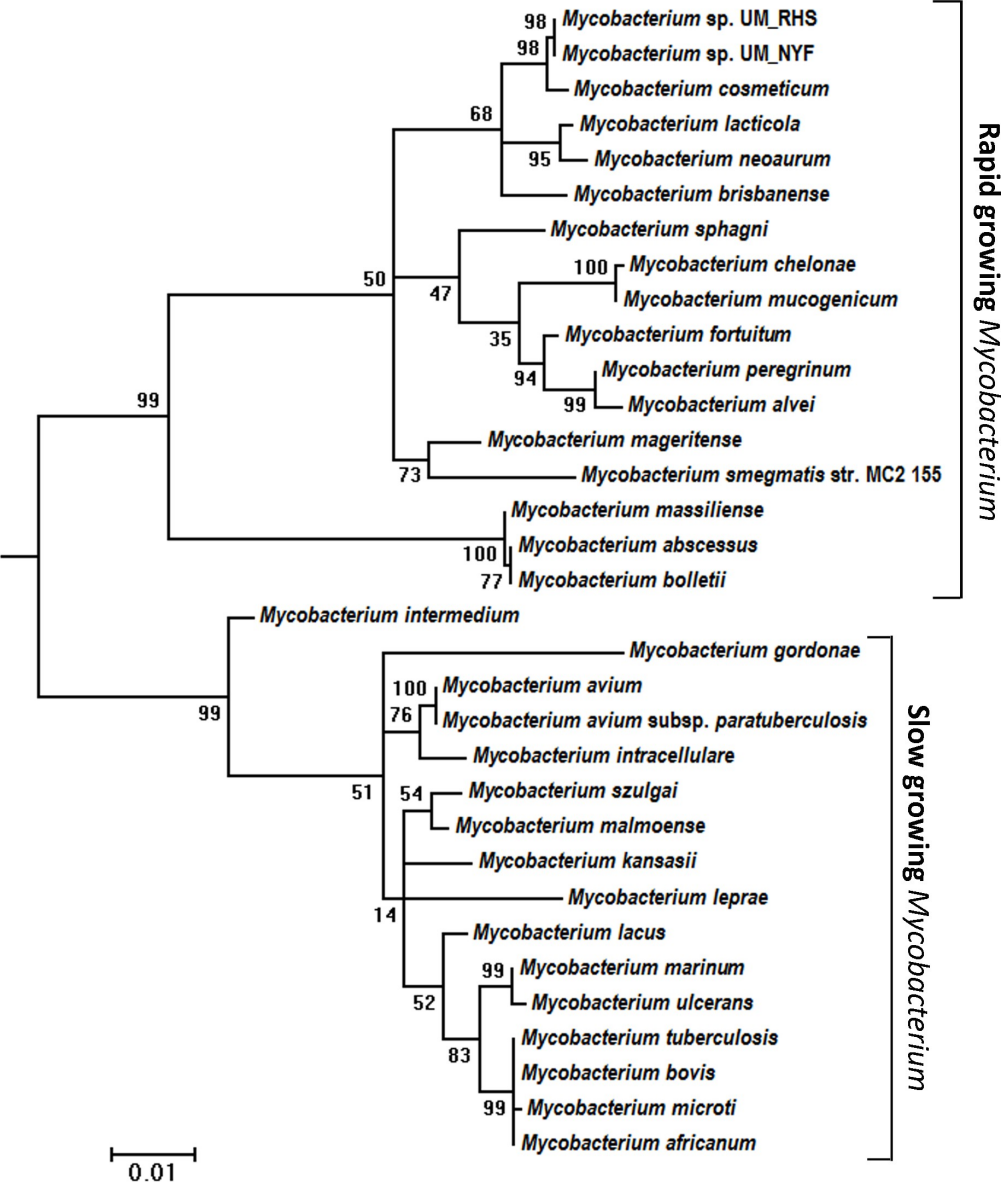
16S rRNA based phylogeny analysis of 32 Mycobacteria strains belonging to different species: Differentiation into rapid and lowly growing Mycobacteria. The *16S rRNA* gene-based phylogenetic tree clearly distinguishes *Mycobacterium* grouping based on the growth rates. UM_RHS and UM_ NYF were clustered in the rapid growing mycobacterial group.

To determine the taxonomic positions of the UM_RHS and UM_NYF, we first constructed phylogenetic trees using housekeeping genes. We constructed a 16S rRNA-based phylogenetic tree using UM_RHS, UM_NYF and other mycobacterial species (Fig 1). Our data suggested that our strains were closely related to *M*. *cosmeticum* with four mismatches and 99% sequence similarities to the reference *M*. *cosmeticum* DSM44829. The *16S rRNA* gene-based tree has been used to separate between the rapid and slow growing mycobacteria [17]. As anticipated, our data clearly separated the two distinct groups and suggested that both UM_RHS and UM_NYF are rapid growing mycobacteria (Fig 1).

**Fig 2:**
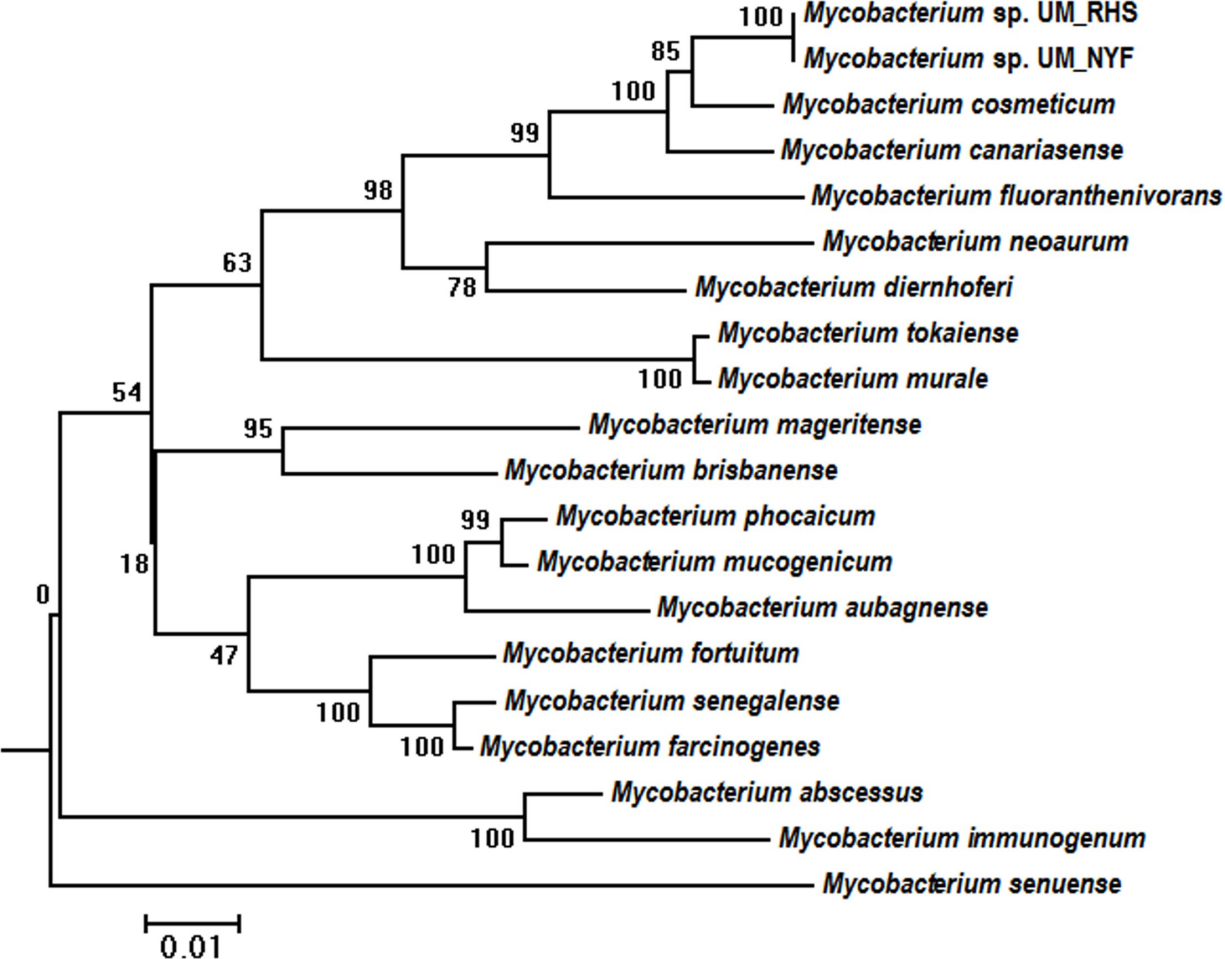
Supermatrix tree of five markers (*hsp65*, *rpoB*, *tuf*, *sodA* and *16S rRNA*). UM_RHS and UM_NYF are closest to *M*. *cosmeticum*, supported by a high bootstrap value of 85%.

The possibility of our strains being *M*. *cosmeticum* is further supported by a supermatrix tree constructed using multiple genes: *hsp65*, *rpoB*, *tuf*, *sodA* and *16S rRNA*. This approach would produce more robust tree compared to the single gene approach (Fig 2) [18]. Our supermatrix tree showed that both UM_RHS and UM_NYF shared highest genome similarities (98.0%) with the reference *M*. *cosmeticum* strain, again acting as an evidence that UM_RHS and UM_NYF are likely *M*. *cosmeticum*.

To further confirm the identity of UM_RHS and UM_NYF, we also performed ANI analysis using whole-genome data. The ANI is one of the most robust measurements of genomic relatedness between bacterial strains [19], and has a great potential in the taxonomy classification of bacteria as a substitute for the traditional labor intensive DNA-DNA hybridization (DDH) technique. The algorithm designed is based on the calculation of average percentage of whole-genome sequence similarity between a pair of bacterial genome. An ANI threshold of 95% determined for species demarcation has previously been suggested based on intensive comparative investigations [19].

To calculate the ANI values (in percentage), we compared the UM_RHS and UM_NYF separately to other 35 *Mycobacterium* species (representative strains for known *Mycobacterium* species) that have genome sequences available in the National Center for Biotechnology Information (NCBI) GenBank depository.

**Fig 3:**
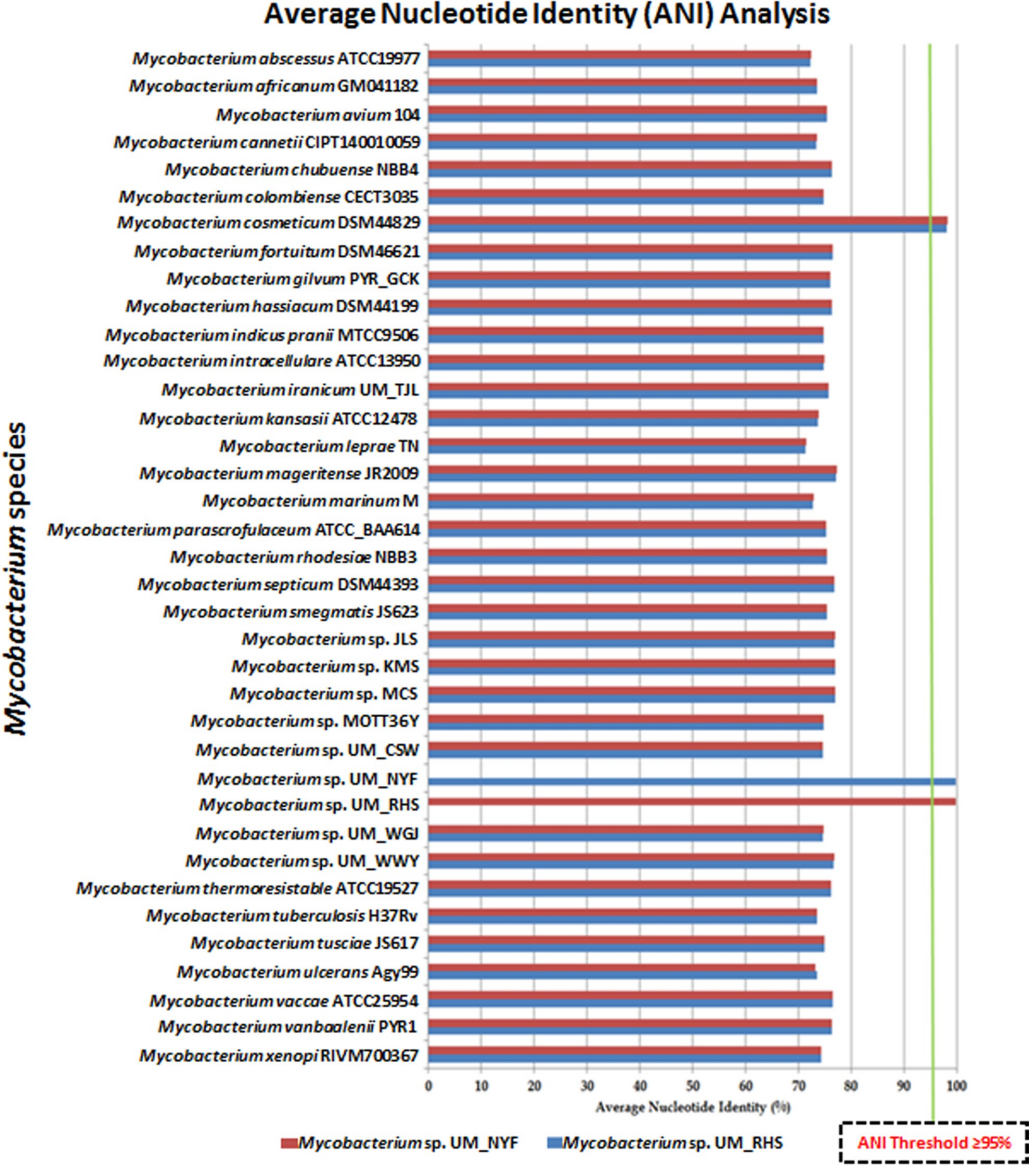
Average nucleotide analysis for 37 *Mycobacterium* species. The ANI values of UM_NYF and UM_RHS against *M*. *cosmeticum* DSM44829 are above 95%, supporting that these strains belong to the species *M*. *cosmeticum*.

The ANI values of each pairwise genome comparison varied from 70% to 99% of sequence identity (Fig 3). The ANI values of UM_RHS and UM_NYF against other 34 strains (excluded the reference strain of *M*. *cosmeticum* DSM44829) ranged from 70% to 78% (below the cut-off to define a species), suggesting that our strains do not belong to these known species. However, when comparing against the reference *M*. *cosmeticum* DSM44829, the UM_RHS and UM_NYF have ANI values of 98.14% and 98.16% respectively, again supporting our view that both UM_RHS and UM_NYF are likely *M*. *cosmeticum*.

Here we have successfully sequenced and analyzed the genomes of probable two new members of *M*. *cosmeticum*, UM_RHS and UM_NYF, which were isolated from patients in Indonesia and Malaysia respectively. The taxonomic position of the two UM_RHS and UM_NYF has been supported by evidence from phylogenetic and ANI analysis.

### Gene prediction and annotation

**Fig 4:**
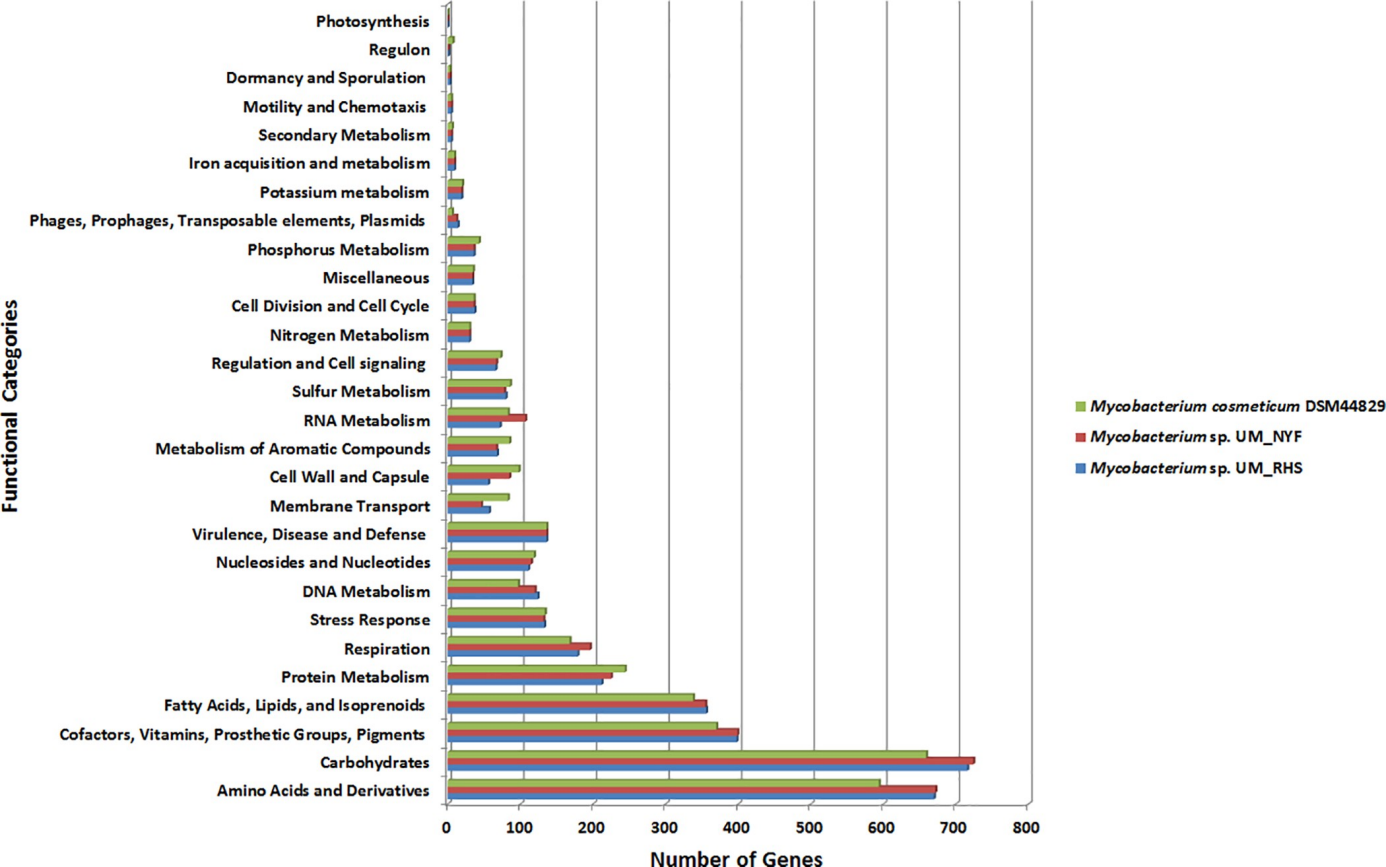
RAST functional categories of UM_RHS and UM_NYF genes. Number of genes in UM_RHS in comparison to UM_NYF which belong to specific RAST functional categories with *M*. *cosmeticum* DSM 44829 as the reference genome.

As anticipated, both UM_RHS and UM_NYF genomes generally share similar genomic features such as the genome size, number of protein-coding genes and RNA as predicted by the Rapid Annotation using Subsystem Technology (RAST) pipeline [20]. For instance, the UM_RHS has a genome size of 6,780,714bp with 6,608 protein-coding genes and 49 RNA genes, whereas UM_NYF has a genome size of 6,809,253bp with 6,579 protein-coding genes and 50 RNA genes (Table 1). Both genomes have single copy of rRNA operons. The summary of functional assignments of the RAST-predicted protein-coding genes for the UM_RHS, UM_NYF and *M*. *cosmeticum* DSM44829 are shown in Fig 4. As anticipated, all the 3 genomes generally share very similar functional distributions since they are most probably belonging to the same species.

**Table 1:** RNAs identified by RAST in the genomes of UM_NYF and UM_RHS.

	UM_NYF	RAST Feature ID	UM_RHS	RAST Feature ID
1	tRNA-Leu-TAA	fig|6666666.28483.rna.1	tRNA-Pro-GGG	fig|6666666.28480.rna.1
2	tRNA-Ile-GAT	fig|6666666.28483.rna.2	tRNA-Asn-GTT	fig|6666666.28480.rna.2
3	tRNA-Ala-TGC	fig|6666666.28483.rna.3	tRNA-Lys-CTT	fig|6666666.28480.rna.3
4	tRNA-Leu-CAG	fig|6666666.28483.rna.4	tRNA-Arg-CCG	fig|6666666.28480.rna.4
5	tRNA-Pro-TGG	fig|6666666.28483.rna.5	tRNA-Thr-CGT	fig|6666666.28480.rna.5
6	tRNA-Gly-TCC	fig|6666666.28483.rna.6	tRNA-Tyr-GTA	fig|6666666.28480.rna.6
7	tRNA-Trp-CCA	fig|6666666.28483.rna.7	tRNA-Met-CAT	fig|6666666.28480.rna.7
8	tRNA-Met-CAT	fig|6666666.28483.rna.8	tRNA-Met-CAT	fig|6666666.28480.rna.8
9	tRNA-Thr-GGT	fig|6666666.28483.rna.9	tRNA-Ala-GGC	fig|6666666.28480.rna.9
10	tRNA-Arg-ACG	fig|6666666.28483.rna.10	tRNA-Val-GAC	fig|6666666.28480.rna.10
11	tRNA-Pseudo-GCT	fig|6666666.28483.rna.11	tRNA-Cys-GCA	fig|6666666.28480.rna.11
12	tRNA-Ser-TGA	fig|6666666.28483.rna.12	tRNA-Gly-GCC	fig|6666666.28480.rna.12
13	tRNA-His-GTG	fig|6666666.28483.rna.13	tRNA-Val-CAC	fig|6666666.28480.rna.13
14	tRNA-Tyr-GTA	fig|6666666.28483.rna.14	5S RNA	fig|6666666.28480.rna.14
15	tRNA-Gln-CTG	fig|6666666.28483.rna.15	Large Subunit Ribosomal RNA; lsuRNA; LSU rRNA	fig|6666666.28480.rna.15
16	tRNA-Glu-CTC	fig|6666666.28483.rna.16	Small Subunit Ribosomal RNA; ssuRNA; SSU rRNA	fig|6666666.28480.rna.16
17	tRNA-Ala-CGC	fig|6666666.28483.rna.17	tRNA-Cys-GCA	fig|6666666.28480.rna.17
18	tRNA-Met-CAT	fig|6666666.28483.rna.18	tRNA-Leu-CAA	fig|6666666.28480.rna.18
19	tRNA-Gln-TTG	fig|6666666.28483.rna.19	tRNA-Leu-TAG	fig|6666666.28480.rna.19
20	tRNA-Val-TAC	fig|6666666.28483.rna.20	tRNA-Ser-GGA	fig|6666666.28480.rna.20
21	tRNA-Lys-CTT	fig|6666666.28483.rna.21	tRNA-Ser-CGA	fig|6666666.28480.rna.21
22	tRNA-Met-CAT	fig|6666666.28483.rna.22	tRNA-Pro-TGG	fig|6666666.28480.rna.22
23	5S RNA	fig|6666666.28483.rna.23	tRNA-Gly-TCC	fig|6666666.28480.rna.23
24	Large Subunit Ribosomal RNA; lsuRNA; LSU rRNA	fig|6666666.28483.rna.24	tRNA-Arg-TCT	fig|6666666.28480.rna.24
25	Small Subunit Ribosomal RNA; ssuRNA; SSU rRNA	fig|6666666.28483.rna.25	tRNA-Ile-GAT	fig|6666666.28480.rna.25
26	tRNA-Leu-GAG	fig|6666666.28483.rna.26	tRNA-Ala-TGC	fig|6666666.28480.rna.26
27	tRNA-Leu-CAA	fig|6666666.28483.rna.27	tRNA-Leu-TAA	fig|6666666.28480.rna.27
28	tRNA-Thr-TGT	fig|6666666.28483.rna.28	tRNA-Phe-GAA	fig|6666666.28480.rna.28
29	tRNA-Ala-GGC	fig|6666666.28483.rna.29	tRNA-Asp-GTC	fig|6666666.28480.rna.29
30	tRNA-Asn-GTT	fig|6666666.28483.rna.30	tRNA-Glu-TTC	fig|6666666.28480.rna.30
31	tRNA-Thr-CGT	fig|6666666.28483.rna.31	tRNA-Lys-TTT	fig|6666666.28480.rna.31
32	tRNA-Ser-CGA	fig|6666666.28483.rna.32	tRNA-Arg-CCT	fig|6666666.28480.rna.32
33	tRNA-Ser-GGA	fig|6666666.28483.rna.33	tRNA-His-GTG	fig|6666666.28480.rna.33
34	tRNA-Arg-CCT	fig|6666666.28483.rna.34	tRNA-Leu-GAG	fig|6666666.28480.rna.34
35	tRNA-Leu-CAA	fig|6666666.28483.rna.35	tRNA-Thr-TGT	fig|6666666.28480.rna.35
36	tRNA-Cys-GCA	fig|6666666.28483.rna.36	tRNA-Ser-TGA	fig|6666666.28480.rna.36
37	tRNA-Gly-CCC	fig|6666666.28483.rna.37	tRNA-Pseudo-GCT	fig|6666666.28480.rna.37
38	tRNA-Arg-CCG	fig|6666666.28483.rna.38	tRNA-Arg-ACG	fig|6666666.28480.rna.38
39	tRNA-Pro-GGG	fig|6666666.28483.rna.39	tRNA-Thr-GGT	fig|6666666.28480.rna.39
40	tRNA-Leu-TAG	fig|6666666.28483.rna.40	tRNA-Met-CAT	fig|6666666.28480.rna.40
41	tRNA-Pro-CGG	fig|6666666.28483.rna.41	tRNA-Trp-CCA	fig|6666666.28480.rna.41
42	tRNA-Val-CAC	fig|6666666.28483.rna.42	tRNA-Val-TAC	fig|6666666.28480.rna.42
43	tRNA-Gly-GCC	fig|6666666.28483.rna.43	tRNA-Ala-CGC	fig|6666666.28480.rna.43
44	tRNA-Cys-GCA	fig|6666666.28483.rna.44	tRNA-Glu-CTC	fig|6666666.28480.rna.44
45	tRNA-Val-GAC	fig|6666666.28483.rna.45	tRNA-Gln-CTG	fig|6666666.28480.rna.45
46	tRNA-Arg-TCT	fig|6666666.28483.rna.46	tRNA-Pro-CGG	fig|6666666.28480.rna.46
47	tRNA-Lys-TTT	fig|6666666.28483.rna.47	tRNA-Gly-CCC	fig|6666666.28480.rna.47
48	tRNA-Glu-TTC	fig|6666666.28483.rna.48	tRNA-Gln-TTG	fig|6666666.28480.rna.48
49	tRNA-Asp-GTC	fig|6666666.28483.rna.49	tRNA-Leu-CAG	fig|6666666.28480.rna.49
50	tRNA-Phe-GAA	fig|6666666.28483.rna.50		

Functional annotation showed that most of the genes were involved in functional categories such as amino acid and derivatives, carbohydrates, cofactors, vitamins, fatty acids, lipids and isoprenoids, which are responsible for basic functions of bacteria. No plasmids were predicted in either of the genomes. RAST predicted the presence of a number of genes encoding integrase, transposase like proteins, phage like proteins and mobile element proteins in both genomes (Table 2). There are about 136 genes categorized under the virulence, disease and defence.

**Table 2:** RAST predicted genes related to gene transfer in the genomes of UM_NYF and UM_RHS.

Types of genes encoding	UM_RHS	UM_NYF
Integrase	4	4
Mobile element proteins	37	34
Phage like proteins	18	18
Transposases like proteins	6	3

However, there are subtle differences between the UM_RHS and UM_NYF in the categories of RNA metabolism, and cell wall and capsule. For instance, UM_NYF genome has 107 genes involved in the RNA metabolism, which is relatively higher compared to the UM_RHS genome (72 genes) (S1 Table and S2 Table). Interestingly, further examination of these genes revealed that UM_NYF possessed many genes in two extra sub-categories: tRNA modification bacteria (26 genes) and *16S rRNA* modification within the P site of its ribosome (6 genes), which were absent in UM_RHS. Out of these six genes categorized under the *16S rRNA* modification within the P site of its ribosome, we identified two methyltransferases, *RsmH* and *RsmI* responsible for the *N*^4^-methylation and 2’-*O*-methylation in the 16S rRNA [21]. These two genes can stabilize the local structure and interaction of the ribosome P-site to accommodate the codon-anticodon helix^21^. Kimura and Suzuki showed that deletions of *rsmH* or *rsmI* can affect the efficiency of non-AUG initiation and the fidelity of translation. Thus, the absence of these two genes in UM_RHS could affect its decoding fidelity [21].

### Comparative genome analysis of *M*. *cosmeticum*

**Fig 5:**
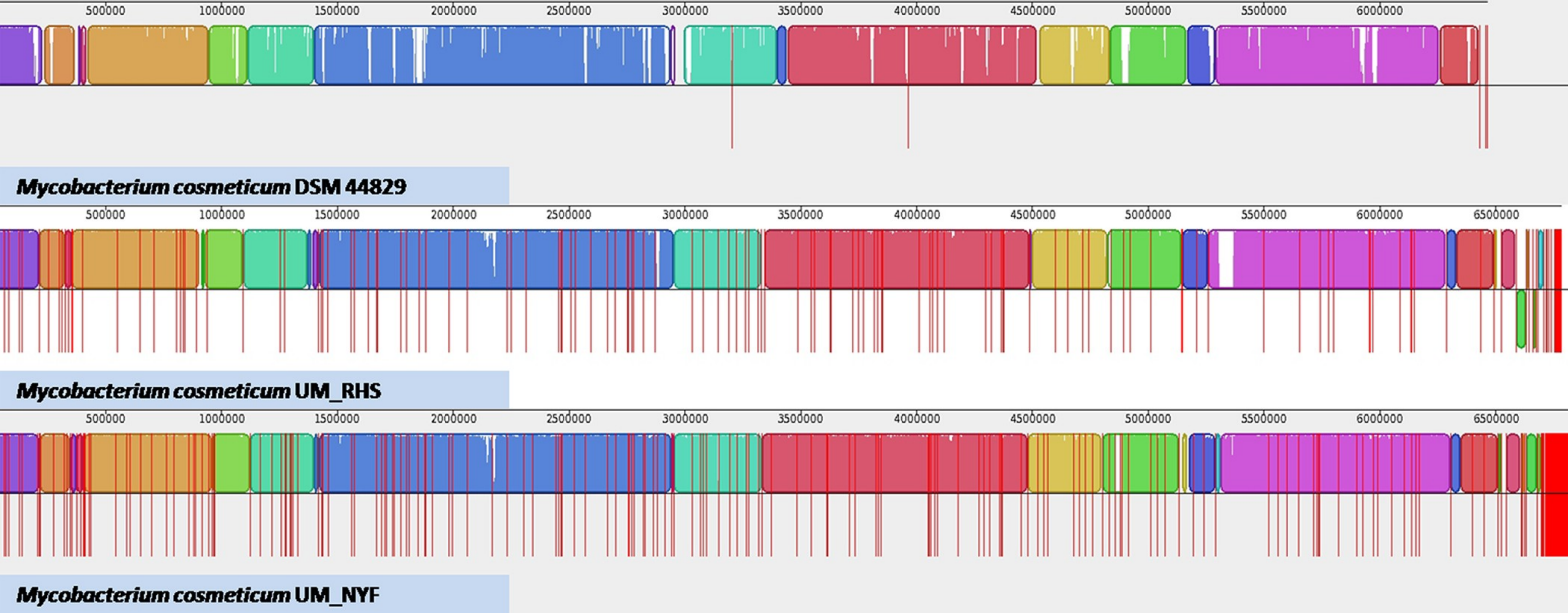
Genomic structure of *M*. *cosmeticum* genomes. The genome structures are generally conserved among three studied genomes (UM_RHS, UM_NYF and DSM44829).

To better understand the genomic structure of *M*. *cosmeticum*, we compared UM_RHS and UM_NYF to a reference genome, *M*. *cosmeticum* DSM 44829 at genome and gene levels. At the genome level, we aligned and reordered the genome sequences of the three strains using Mauve software with *M*. *cosmeticum* DSM 44829 as a reference [22]. We found that the three *M*. *cosmeticum* genomes were generally similar or conserved as most of the genome regions for the three strains were nicely aligned to each other (Fig 5). The UM_RHS and UM_NYF mapped approximately 92% of the reference genome with a high sequence identity of 98%. However, the genome size of UM_RHS and UM_NYF was comparatively larger (~0.4Mbp) than the reference genome which has a genome size of about 6.4Mbp. Both UM_RHS and UM_NYF have larger genome size probably due to the presence of considerably large number of horizontally transferred genomic islands found in both genomes which we will discussed below.

**Fig 6:**
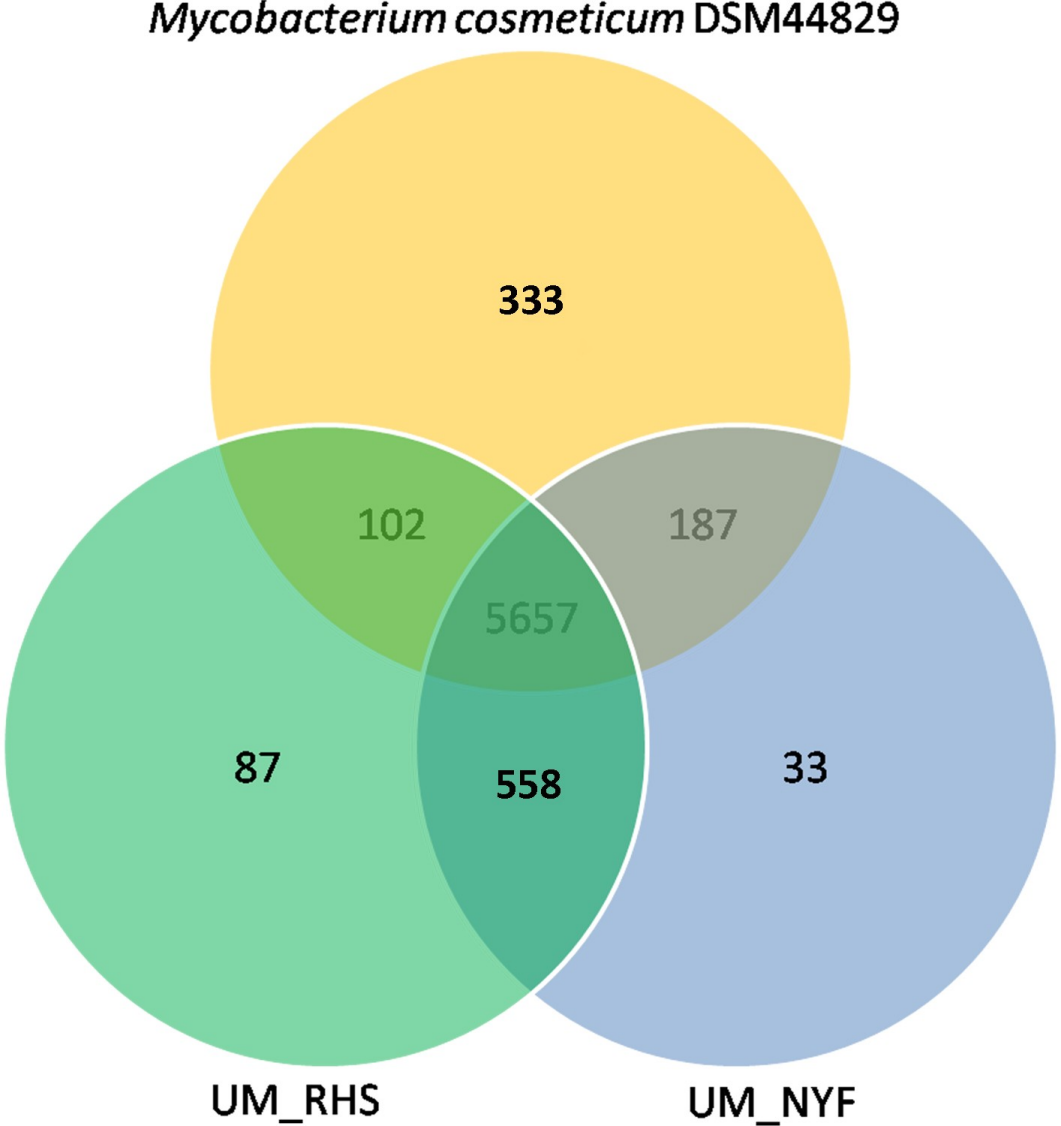
Gene family distribution. The *M*. *cosmeticum* DSM 44829, UM_RHS and UM_NYF have generally shared a high number of common gene families.

At the gene level, we clustered all RAST-predicted genes of the three strains using BLASTClust (http://ftp.ncbi.nih.gov/blast/documents/blastclust.html) which resulted in non-redundant 6,957 orthologous gene families. Our data clearly showed that the three strains shared a high number of common gene families (5,657), which accounted for 81.3% of the total gene families suggesting that they are considerably conserved among them (Fig 6). Interestingly, we found a relatively higher number of strain-specific gene families (333) in DSM 44829 compared to UM_RHS (87) and UM_NYF (33). Of these 333 genes, 89 (26.7%) were believed to be inserted into the *M*. *cosmeticum* DSM 44829 genome as these genes were found inside the predicted horizontally transferred genomic islands in the *M*. *cosmeticum* DSM 44829. In addition, we also found two putative *M*. *cosmeticum* DSM 44829 specific-genes, Type I restriction modification enzymes that may play important roles in the defense mechanism of the bacteria [23].

Another interesting observation is that 558 gene families were present only in both UM_RHS and UM_NYF but not in the *M*. *cosmeticum* DSM 44829. The high number of shared genes between UM_RHS and UM_NYF is probably due to the horizontal gene transfer supported by the observation that at least 154 of these genes are (28%) found within the predicted genomic islands. These specific genes may have arisen from the ancestors of UM_RHS and UM_NYF and probably contribute to the unique traits/phenotypes of these strains which are absent in the previously reported *M*. *cosmeticum* DSM 44829. Among these specific genes, five specific genes together with other four non-specific genes formed a cluster of nine genes (~12 Kbp) encoding enzymes which metabolize acetone and acetoacetate to acetyl-CoA (Acetone utilization pathway) [24]. Similar gene cluster has also been found in *Helicobacter pylori* and *Helicobacter acinonychus* strains [24]. However, the *Helicobacter pylori* strains contain only a cluster of eight genes lacking one gene (*acxD*) compared to the UM_RHS and UM_NYF, harboring four *acx* genes (*acxABCD*), *scoA*, *scoB*, *fadA* and two hypothetical proteins. The presence of the gene cluster in the sequenced genomes of UM_RHS and UM_NYF may indicate the capability of these strains to metabolize acetone to acetyl-CoA and feed into the TCA cycle, thus providing them with energy [24].

Furthermore, we wanted to identify genes that are specific to *M*. *cosmeticum*, but not in other *Mycobacterium* species. To examine this, we further clustered the 5,657 common gene families of UM_RHS and UM_NYF with genes from other known mycobacterial genomes belonging to 27 different mycobacterial species. We successfully identified 552 gene families specific to *M*. *cosmeticum* but not present in other mycobacterial species that we examined. Among these specific genes, we found a gene operon involved in the sorbitol (glucitol) specific phosphoenolpyruvate-dependent sugar phosphotransferase (PTS) system, which has three components (EIIA, EIIB and EIIC) [25, 26].

### Comparative genomic islands analysis

To predict horizontally transferred genomic islands, all genome sequences used in this analysis were uploaded to the IslandViewer server [27, 28]. A total of 41 putative genomic islands were found in the two genomes with genomic sizes ranging from 4k to 32kbp (S3 Table). Both strains shared 34 common genomic islands by which five are specific to UM_RHS and two other specific to UM_NYF. A high number of putative genomic islands were present in the two genomes indicating that horizontal gene transfer events might have played significant roles in reshaping the genomes of UM_RHS and UM_NYF throughout the evolutionary period. Among these common genomic islands, some have harbored putative virulence genes which could contribute to the virulence of the two strains. For instance, one of the genomic islands (GI23) harbored two putative virulence genes, *espR* and *phoP*. Another genomic island, GI28, has a virulence gene *sigH*.

Furthermore, we found a genomic island which is believed to have originated from *Mycobacterium goodie* X7B being inserted into both UM_RHS and UM_NYF during the evolutionary period. This genomic island contains the *dsz* (dibenzothiophene biodesulfurization) operon harboring three desulfurization genes (*dszA*, *dszB*, *dszC)*. Combustion of sulfur-containing compounds can cause adverse effects on health and environment [29]. Benzothiophene (BTH) and dibenzothiophene (DBT) account for more than 50% of the sulfur content of diesel. Current industries usually apply hydro-desulfurization which requires high temperature and pressure. However, biodesulfurization is an environmental friendly method to eliminate sulfur from the refractory organic compound. The major pathway of DBT desulfurization has been reported as the “4S pathways”. The “4S pathway” includes four steps which will catalyze DBT into sulfoxide (DBTO), sulfone (DBTO_2_), sulfinate (HPBSi) and hydroxybiphenyl (HBP). These catalytic reactions are carried out by three Dsz enzymes namely DszA, DszB and DszC encoded by the *dsz* operon. Thus, we postulate that the acquisition of this genomic island possibly from *M*. *goodie* X7B might have given both UM_RHS and UM_NYF the capability to catalyze the DBT desulfurization.

Besides, three large common genomic islands associated with prophages were identified by screening the genomic regions using PHAST software, a software to predict prophage sequences in bacterial genomes or plasmids [30]. As predicted by PHAST, two are intact prophages and the remaining one is an incomplete prophage in the genomes of UM_RHS and UM_NYF. However, the origin of these prophages are yet to be identified as no hit could be found from BLAST search of these sequences against the NCBI databases.

### Virulence gene analyses

**Fig 7:**
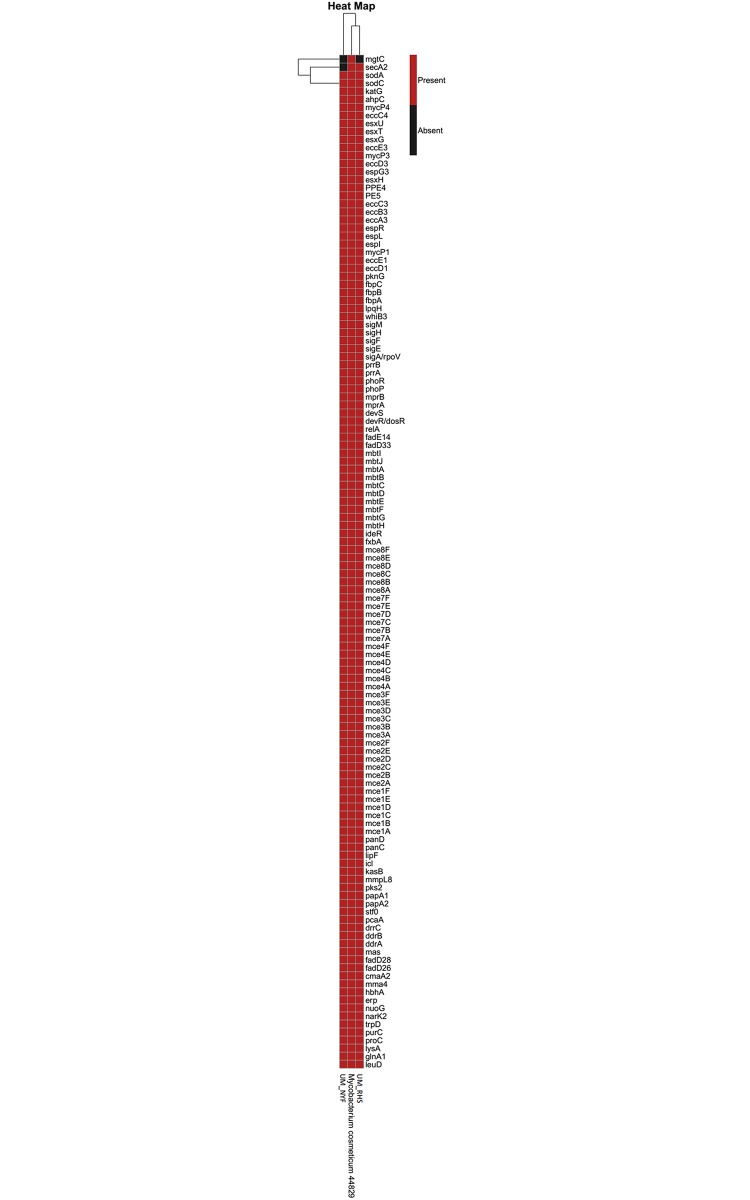
Predicted virulence genes in the genomes of *M*. *cosmeticum* DSM44829, UM_RHS and UM_NYF.

Our analysis revealed that both UM_RHS and UM_NYF share 117 similar putative virulence genes with UM_RHS having one extra virulence gene (*secA*) resulting in a total of 118 putative virulence genes in UM_RHS and 117 in UM_NYF (Fig 7). Furthermore, most of the virulence genes found in both genomes were orthologs to well-known human pathogens like *M*. *tuberculosis*. Gey van Pittius and colleagues have shown that the *ESX-5* genes cluster, a type VII secretion system, is able to separate the rapid and slow growing mycobacteria. They showed that this *ESX-5* genes cluster is only present in the slow growing mycobacteria [31]. Likewise, our strains UM_RHS and UM_NYF do not have the ESX-5 gene cluster, further supporting that the two strains are rapid growing mycobacteria and this is also consistent with the results from the phylogenetic analysis. The data showed that UM_RHS and UM_NYF contain three *ESX* gene clusters (ESX1, 3, 4) although a few genes were missing in these clusters probably due to gene deletion events. However, since both UM_RHS and UM_NYF are draft genomes, additional experiments e.g. PCR are crucial to further confirm the existence of these genes.

Apart from the *ESX* gene clusters, both UM_RHS and UM_NYF genomes showed presence of other putative virulence genes such as *ahpC*, *katG*, *sodC* responsible for enhancing resistance against host toxic compounds, whereas *nuoG* and *sodA* which are involved in evading apoptosis [32]. Some of these genes (*ahpC*, *katG* and *sodC*) encode enzymes important for detoxification of bacteria killing component like reactive oxygen species (ROS) and reactive nitrogen species (RNS), wheareas the *nuoG* gene encodes for protein involving the inhibition of extrinsic TNF-α- dependent apoptosis pathway [33]. Furthermore, we also identified virulence genes (*fbp*, *erp*, *hbhA*, *mce*) which encode for cell envelope proteins [34]. These proteins are important in the mycobacterial cell wall maintenance, adhesion and transportation of materials and also survival of mycobacteria in the host cells [34]. Both UM_RHS and UM_NYF also harbored three *fbp* genes (*fbpA*, *fbpB* and *fbpC*) encoding for the antigen 85 complex [34]. This complex is known to be related to the pathogenesis of mycobacteria, for example, by promoting the entry of bacteria into host cells through the binding of fibronectin [35].

In order to survive in host cells, mycobacteria have to adapt to a wide range of environments, stressors and growth condition [34]. Two component regulatory signal transduction systems are important in allowing bacteria to adapt to a variety of environmental stimuli. Interestingly, four pairs of two-component systems were found (PhoP-PhoR, DevR-DevS, MprA-MprB and PrrA-PrrB) in the genomes of UM_RHS and UM_NYF. Previous studies have demonstrated that disruption of the *phoP-phoR* operon can affect the replication of *M*. *tuberculosis* in the cellular and animal models [36, 37]. This gene operon is also involved in the regulation of genes with potential roles in mycobacterial virulence by regulating the expression of both *espB* and *espR* genes in the ESX-1 secretion system [38].

Overall, both genomes are structurally conserved although differences in the genomic islands and virulence genes of these genomes can be observed. We did not notice any large genome inversions in any of the *M*. *cosmeticum* strains. The number of shared genes between UM_RHS and UM_NYF were higher compared to that of UM_RHS and *M*. *cosmeticum* DSM44829 or UM_NYF and *M*. *cosmeticum* DSM44829, probably reflecting the fact that both UM_RHS and UM_NYF are highly similar because they were isolated from very close geographical regions (Indonesia and Malaysia respectively), whereas the *M*. *cosmeticum* DSM44829 was isolated from a granulomatous lesion of a female patient in Venezuela [12] [https://www.dsmz.de/catalogues/details/culture/DSM-44829.html].

Both the genomes of UM_RHS and UM_NYF showed presence of sorbitol (glucitol) specific phosphoenolpyruvate-dependent sugar phosphotransferase (PTS) system [25, 26]. Sorbitol is a sugar substitute used when caries formation occurs in the presence of readily fermentable carbohydrate like sucroses. We hypothesize that in the presence of these genes, UM_RHS and UM_NYF could be capable of utilizing sugars using the PTS system.

One interesting finding in this study is the presence of *dsz* (dibenzothiophene biodesulfurization) operon in the horizontally transferred genomic island in the genomes of UM_RHS and UM_NYF. Our data suggested that this genomic region might have originated from *M*. *goodie* X7B, which has the capability to desulfurize benzothiophene (BTH) and dibenzothiophene (DBT) through the BTH degradation pathway [39]. Combustion of fossil fuel such as petroleum releases sulfur oxides causing air pollution [40, 41]. Deep desulfurization of gasoline can reduce sulfur content, but the conventional hydrodesulfurization (HDS) technology of gasoline results in a significant reduction of the octane number [42]. The biodesulfization of gasoline can serve as an alternative method of sources which not only avoids octane degradation but is also less expensive as compared to the HDS method [39]. Therefore, we postulate that the UM_RHS and UM_NYF may be able to be used for performing biodesulfization of gasoline or with its additionally potential to reduce the expenses and air pollution during fuel combustion. However, further experiments are needed to confirm the usability of this bacterial species for these purposes.

In comparison to the reference genome, *M*. *cosmeticum* DSM44829, we found relatively higher number of genes and genomic islands in the genomes of UM_RHS and UM_NYF. Intriguingly, our comparative analysis revealed two genes encoding for Type I restriction modification enzymes in *M*. *cosmeticum* DSM44829, whereas the UM_RHS and UM_NYF lack these genes that are important for bacterial defense. Therefore, the presence of high number of genomic islands in our strains may be partially explained by the fact that they might be more prone to invasion by foreign DNA compared to the reference strain.

In summary, the phylogenetic and ANI analysis of these two investigated strains UM_RHS and UM_NYF showed that they most likely belong to the species *M*. *cosmeticum*. The addition of these genome sequences may be an important avenue for comparative analyses and functional studies of *M*. *cosmeticum* in future.

## Methods

### Library construction and next-generation sequencing

The DNA of UM_RHS and UM_NYF were sequenced using Illumina HiSeq 2000 PE technology at about 1,000X coverage. Covaris S2 was used to fragment the DNA samples for 120 seconds at a temperature of 5.5–6.0 degree celcius. The quantity and quality of fragmented materials were examined using Agilent BioAnalyzer 2100. The sample was size selected using Invitrogen 2% agarose E-gels. Fragments with adapter molecules at both ends further underwent 10 cycles of PCR for library construction purpose. Agilent BioAnalyzer 2100 was used to validate the constructed genomic library and a pool of 8pM was loaded onto 1 lane of Illumina HiSeq2000 flow cell v3 for sequencing using a 100bp PE sequencing strategy.

### Read preprocessing and genome assembly

PRINSEQ lite version 0.20 [16] was used to filter exact duplicates and reverse complement duplicate reads. Reads were trimmed at Phred quality score < 20. The final reads were *de novo* assembled using CLC Genomic Workbench version 5.1 (CLC bio, Aarhus, Denmark). The assembly of the preprocessed reads were performed using the following criteria: length fraction of 0.7, similarity fraction of 0.9, and any contigs with size lesser than 500bp were discarded.

### Phylogenetic inferences

The *16S rRNA*-based phylogenetic tree was constructed using Hasegawa-Kishino-Yano DNA substitution model with a bootstrap value of 500. Five selected bacterial classification marker genes, *hsp65*, *rpoB*, *tuf*, *sodA* and *16S rRNA* from the closest species were extracted and concatenated for construction of supermatrix-based tree using the same approach as the 16S rRNA-based tree.

### Genome annotation

The genome of UM_RHS and UM_NYF were annotated using the RAST annotation pipeline [20]. To predict the genomic islands, the assembled sequences of UM_RHS and UM_NYF were submitted to IslandViewer [27, 28]. The generated output results were further filtered by eliminating the genomic islands situated within two different contigs [43]. The RAST-predicted protein coding genes found in the genomes of UM_RHS and UM_NYF were used for virulence genes prediction. BLAST search was performed with the RAST-predicted protein sequences against the Virulence Factors Database (VFDB) [44–46] with e-value of 10 and orthologous genes that have at least 50% sequence identity and 50% sequence completeness and with known virulence genes were considered as putative virulence genes.

### Gene family clustering

The *M*. *cosmeticum* strain DSM44829 (accession no. GCA_000613185.1) isolated from a granulomatous lesion of a female patient in Venezuela [12] [https://www.dsmz.de/catalogues/details/culture/DSM-44829.html], was used as the reference genome sequence for the gene family clustering study. The RAST-predicted protein sequences of UM_RHS, UM_NYF and *M*. *cosmeticum* DSM44829 were clustered into orthologous gene families using BLASTClust with maximal e-value of 1e^-10^ and minimum score of 40 (http://ftp.ncbi.nih.gov/blast/documents/blastclust.html). Protein sequences with at least 50% sequence identity and 50% sequence coverage between each other were clustered into the same orthologous gene family.

### Genomic islands and virulence gene prediction

Genomic islands in UM_RHS and UM_NYF were predicted using IslandViewer [27, 28] with the integration of few approaches such as the sequence composition based SIGI-HMM [47] and IslandPath-DIMOB [48] and the comparative genomics approach IslandPick [28]. The generated results from IslandViewer were further filtered by eliminating the islands situated within 2 contigs. The RAST-predicted protein sequences in UM_RHS and UM_NYF were further BLAST searched against the Virulence Factors Database (VFDB). The BLAST results were filtered using in-house Perl scripts to select orthologous genes that are at least 50% sequence identity and 50% sequence completeness.

## Supporting information

S1 Table RNAs identified by RAST in the genomes of UM_NYF and UM_RHS. (DOCX)

S2 Table RAST predicted genes related to gene transfer in the genomes of UM_NYF and UM_RHS. (DOCX)

S3 Table Genomic islands present in UM_NYF and UM_RHS. (DOCX)

S1 Fig The graphical representation of GI35. (PDF)
