# Supplementary material for: Comparative genome sequencing and analyses of Mycobacterium cosmeticum reveal potential for biodesulfization of gasoline
Source: PLoS One. 2019 Apr 9;14(4):e0214663. doi: 10.1371/journal.pone.0214663 (PMC6456199; doi:10.1371/journal.pone.0214663)
Supplement: S1 Table — (DOCX) [file pone.0214663.s001.docx]

**S1 Table. RNAs identified by RAST in the genomes of UM_NYF and UM_RHS.**

|  | **Category** | **Subcategory** | **Subsystem** | **Role** | **Features** |
| --- | --- | --- | --- | --- | --- |
| 1 | RNA Metabolism | RNA processing and modification | RNA pseudouridine syntheses | Ribosomal large subunit pseudouridine synthase A (EC 4.2.1.70) | fig\|6666666.31767.peg.4499 |
| 2 | RNA Metabolism | RNA processing and modification | RNA pseudouridine syntheses | Ribosomal large subunit pseudouridine synthase D (EC 4.2.1.70) | fig\|6666666.31767.peg.4680 |
| 3 | RNA Metabolism | RNA processing and modification | RNA pseudouridine syntheses | Ribosomal large subunit pseudouridine synthase B (EC 4.2.1.70) | fig\|6666666.31767.peg.19 |
| 4 | RNA Metabolism | RNA processing and modification | RNA pseudouridine syntheses | tRNA pseudouridine synthase B (EC 4.2.1.70) | fig\|6666666.31767.peg.4897 |
| 5 | RNA Metabolism | RNA processing and modification | RNA pseudouridine syntheses | tRNA pseudouridine synthase A (EC 4.2.1.70) | fig\|6666666.31767.peg.2553 |
| 6 | RNA Metabolism | RNA processing and modification | tRNA nucleotidyltransferase | tRNA nucleotidyltransferase (EC 2.7.7.21) (EC 2.7.7.25) | fig\|6666666.31767.peg.2770 |
| 7 | RNA Metabolism | RNA processing and modification | Methylthiotransferases | tRNA-i(6)A37 methylthiotransferase | fig\|6666666.31767.peg.241 |
| 8 | RNA Metabolism | RNA processing and modification | RNA 3'-terminal phosphate cyclase | RNA 3'-terminal phosphate cyclase (EC 6.5.1.4) | fig\|6666666.31767.peg.4010 |
| 9 | RNA Metabolism | RNA processing and modification | RNA processing and degradation, bacterial | FIG146085: 3'-to-5' oligoribonuclease A, Bacillus type | fig\|6666666.31767.peg.4918 |
| 10 | RNA Metabolism | RNA processing and modification | RNA processing and degradation, bacterial | Ribonuclease III (EC 3.1.26.3) | fig\|6666666.31767.peg.5460 |
| 11 | RNA Metabolism | RNA processing and modification | RNA processing and degradation, bacterial | Ribonuclease E inhibitor RraA | fig\|6666666.31767.peg.1070 |
| 12 | RNA Metabolism | RNA processing and modification | RNA processing and degradation, bacterial | Ribonuclease E (EC 3.1.26.12) | fig\|6666666.31767.peg.3523 |
| 13 | RNA Metabolism | RNA processing and modification | RNA processing and degradation, bacterial | 3'-to-5' oligoribonuclease (orn) | fig\|6666666.31767.peg.4059 |
| 14 | RNA Metabolism | RNA processing and modification | RNA methylation | Ribosomal RNA small subunit methyltransferase E (EC 2.1.1.-) | fig\|6666666.31767.peg.3952 |
| 15 | RNA Metabolism | RNA processing and modification | RNA methylation | 23S rRNA (Uracil-5-) -methyltransferase RumA (EC 2.1.1.-) | fig\|6666666.31767.peg.1513 |
| 16 | RNA Metabolism | RNA processing and modification | RNA methylation | SSU rRNA (adenine(1518)-N(6)/adenine(1519)-N(6))-dimethyltransferase (EC 2.1.1.182) | fig\|6666666.31767.peg.6443 |
| 17 | RNA Metabolism | RNA processing and modification | RNA methylation | 23S rRNA (guanosine-2'-O-) -methyltransferase rlmB (EC 2.1.1.-) | fig\|6666666.31767.peg.1813 |
| 18 | RNA Metabolism | RNA processing and modification | RNA methylation | tRNA (guanine46-N7-)-methyltransferase (EC 2.1.1.33) | fig\|6666666.31767.peg.4883 |
| 19 | RNA Metabolism | RNA processing and modification | RNA methylation | Ribosomal RNA large subunit methyltransferase N (EC 2.1.1.-) | fig\|6666666.31767.peg.3571 |
| 20 | RNA Metabolism | RNA processing and modification | RNA methylation | tRNA (Guanine37-N1) -methyltransferase (EC 2.1.1.31) | fig\|6666666.31767.peg.5433 |
| 21 | RNA Metabolism | RNA processing and modification | RNA methylation | rRNA small subunit 7-methylguanosine (m7G) methyltransferase GidB | fig\|6666666.31767.peg.2782 |
| 22 | RNA Metabolism | RNA processing and modification | RNA methylation | tRNA (cytidine(34)-2'-O)-methyltransferase (EC 2.1.1.207) | fig\|6666666.31767.peg.3130 |
| 23 | RNA Metabolism | RNA processing and modification | RNA methylation | tRNA-specific 2-thiouridylase MnmA | fig\|6666666.31767.peg.5541 |
| 24 | RNA Metabolism | RNA processing and modification | RNA methylation | 16S rRNA (guanine(966)-N(2))-methyltransferase (EC 2.1.1.171) | fig\|6666666.31767.peg.5467 |
| 25 | RNA Metabolism | RNA processing and modification | ATP-dependent RNA helicases, bacterial | Cold-shock DEAD-box protein A | fig\|6666666.31767.peg.5395 |
| 26 | RNA Metabolism | RNA processing and modification | Queuosine-Archaeosine Biosynthesis | Peptidyl-prolyl cis-trans isomerase (EC 5.2.1.8) | fig\|6666666.31767.peg.2811 |
| 27 | RNA Metabolism | RNA processing and modification | Queuosine-Archaeosine Biosynthesis | Peptidyl-prolyl cis-trans isomerase (EC 5.2.1.8) | fig\|6666666.31767.peg.3479 |
| 28 | RNA Metabolism | RNA processing and modification | Queuosine-Archaeosine Biosynthesis | Peptidyl-prolyl cis-trans isomerase (EC 5.2.1.8) | fig\|6666666.31767.peg.3480 |
| 29 | RNA Metabolism | RNA processing and modification | Queuosine-Archaeosine Biosynthesis | Inosine-uridine preferring nucleoside hydrolase (EC 3.2.2.1) | fig\|6666666.31767.peg.6194 |
| 30 | RNA Metabolism | RNA processing and modification | Queuosine-Archaeosine Biosynthesis | GTP cyclohydrolase I (EC 3.5.4.16) type 1 | fig\|6666666.31767.peg.778 |
| 31 | RNA Metabolism | RNA processing and modification | Queuosine-Archaeosine Biosynthesis | Putative preQ0 transporter | fig\|6666666.31767.peg.4276 |
| 32 | RNA Metabolism | RNA processing and modification | Queuosine-Archaeosine Biosynthesis | Permease of the drug/metabolite transporter (DMT) superfamily | fig\|6666666.31767.peg.2559 |
| 33 | RNA Metabolism | RNA processing and modification | Queuosine-Archaeosine Biosynthesis | Permease of the drug/metabolite transporter (DMT) superfamily | fig\|6666666.31767.peg.5943 |
| 34 | RNA Metabolism | RNA processing and modification | Queuosine-Archaeosine Biosynthesis | glutamyl-Q-tRNA synthetase | fig\|6666666.31767.peg.2482 |
| 35 | RNA Metabolism | RNA processing and modification | Queuosine-Archaeosine Biosynthesis | tRNA-guanine transglycosylase (EC 2.4.2.29) | fig\|6666666.31767.peg.2488 |
| 36 | RNA Metabolism | RNA processing and modification | Ribonuclease H | Ribonuclease HI (EC 3.1.26.4) | fig\|6666666.31767.peg.3761 |
| 37 | RNA Metabolism | RNA processing and modification | Ribonuclease H | Ribonuclease HII (EC 3.1.26.4) | fig\|6666666.31767.peg.5429 |
| 38 | RNA Metabolism | RNA processing and modification | Ribonuclease H | Protein often found in Actinomycetes clustered with signal peptidase and/or RNaseHII | fig\|6666666.31767.peg.5428 |
| 39 | RNA Metabolism | RNA processing and modification | tRNA processing | Ribonuclease P protein component (EC 3.1.26.5) | fig\|6666666.31767.peg.2786 |
| 40 | RNA Metabolism | RNA processing and modification | tRNA processing | tRNA-i(6)A37 methylthiotransferase | fig\|6666666.31767.peg.241 |
| 41 | RNA Metabolism | RNA processing and modification | tRNA processing | Ribonuclease D (EC 3.1.26.3) | fig\|6666666.31767.peg.1517 |
| 42 | RNA Metabolism | RNA processing and modification | tRNA processing | tRNA pseudouridine synthase B (EC 4.2.1.70) | fig\|6666666.31767.peg.4897 |
| 43 | RNA Metabolism | RNA processing and modification | tRNA processing | tRNA(Ile)-lysidine synthetase | fig\|6666666.31767.peg.6017 |
| 44 | RNA Metabolism | RNA processing and modification | tRNA processing | tRNA-specific adenosine-34 deaminase (EC 3.5.4.-) | fig\|6666666.31767.peg.1356 |
| 45 | RNA Metabolism | RNA processing and modification | tRNA processing | tRNA-specific adenosine-34 deaminase (EC 3.5.4.-) | fig\|6666666.31767.peg.2498 |
| 46 | RNA Metabolism | RNA processing and modification | tRNA processing | tRNA dimethylallyltransferase (EC 2.5.1.75) | fig\|6666666.31767.peg.1466 |
| 47 | RNA Metabolism | RNA processing and modification | tRNA processing | Ribonuclease PH (EC 2.7.7.56) | fig\|6666666.31767.peg.2324 |
| 48 | RNA Metabolism | RNA processing and modification | tRNA processing | tRNA pseudouridine synthase A (EC 4.2.1.70) | fig\|6666666.31767.peg.2553 |
| 49 | RNA Metabolism | Transcription | Transcription initiation, bacterial sigma factors | RNA polymerase sigma-54 factor RpoN | fig\|6666666.31767.peg.1034 |
| 50 | RNA Metabolism | Transcription | Transcription initiation, bacterial sigma factors | RNA polymerase sigma-54 factor RpoN | fig\|6666666.31767.peg.5226 |
| 51 | RNA Metabolism | Transcription | Transcription initiation, bacterial sigma factors | RNA polymerase sigma factor RpoD | fig\|6666666.31767.peg.1499 |
| 52 | RNA Metabolism | Transcription | Transcription initiation, bacterial sigma factors | RNA polymerase sigma-70 factor | fig\|6666666.31767.peg.3593 |
| 53 | RNA Metabolism | Transcription | Transcription initiation, bacterial sigma factors | RNA polymerase sigma-70 factor | fig\|6666666.31767.peg.3791 |
| 54 | RNA Metabolism | Transcription | Transcription initiation, bacterial sigma factors | RNA polymerase sigma-70 factor | fig\|6666666.31767.peg.5055 |
| 55 | RNA Metabolism | Transcription | Transcription initiation, bacterial sigma factors | RNA polymerase sigma-70 factor | fig\|6666666.31767.peg.5333 |
| 56 | RNA Metabolism | Transcription | Transcription initiation, bacterial sigma factors | RNA polymerase sigma-70 factor | fig\|6666666.31767.peg.6222 |
| 57 | RNA Metabolism | Transcription | Transcription initiation, bacterial sigma factors | RNA polymerase sigma factor RpoE | fig\|6666666.31767.peg.2826 |
| 58 | RNA Metabolism | Transcription | Transcription initiation, bacterial sigma factors | RNA polymerase sigma factor SigB | fig\|6666666.31767.peg.1487 |
| 59 | RNA Metabolism | Transcription | Transcription initiation, bacterial sigma factors | RNA polymerase sigma factor SigB | fig\|6666666.31767.peg.5758 |
| 60 | RNA Metabolism | Transcription | RNA polymerase bacterial | DNA-directed RNA polymerase alpha subunit (EC 2.7.7.6) | fig\|6666666.31767.peg.2551 |
| 61 | RNA Metabolism | Transcription | RNA polymerase bacterial | DNA-directed RNA polymerase beta' subunit (EC 2.7.7.6) | fig\|6666666.31767.peg.5000 |
| 62 | RNA Metabolism | Transcription | RNA polymerase bacterial | DNA-directed RNA polymerase omega subunit (EC 2.7.7.6) | fig\|6666666.31767.peg.3406 |
| 63 | RNA Metabolism | Transcription | RNA polymerase bacterial | DNA-directed RNA polymerase beta subunit (EC 2.7.7.6) | fig\|6666666.31767.peg.4999 |
| 64 | RNA Metabolism | Transcription | Transcription factors bacterial | Transcription termination protein NusB | fig\|6666666.31767.peg.3429 |
| 65 | RNA Metabolism | Transcription | Transcription factors bacterial | Transcription accessory protein (S1 RNA-binding domain) | fig\|6666666.31767.peg.38 |
| 66 | RNA Metabolism | Transcription | Transcription factors bacterial | Transcription elongation factor GreA | fig\|6666666.31767.peg.4134 |
| 67 | RNA Metabolism | Transcription | Transcription factors bacterial | Transcription antitermination protein NusG | fig\|6666666.31767.peg.4975 |
| 68 | RNA Metabolism | Transcription | Transcription factors bacterial | FIG000325: clustered with transcription termination protein NusA | fig\|6666666.31767.peg.3635 |
| 69 | RNA Metabolism | Transcription | Transcription factors bacterial | Transcription-repair coupling factor | fig\|6666666.31767.peg.6465 |
| 70 | RNA Metabolism | Transcription | Rrf2 family transcriptional regulators | Predicted transcriptional regulator of sulfate adenylyltransferase, Rrf2 family | fig\|6666666.31767.peg.459 |
| 71 | RNA Metabolism | Transcription | Rrf2 family transcriptional regulators | Nitrite-sensitive transcriptional repressor NsrR | fig\|6666666.31767.peg.1956 |
| 72 | RNA Metabolism | Transcription | Rrf2 family transcriptional regulators | Nitrite-sensitive transcriptional repressor NsrR | fig\|6666666.31767.peg.4968 |
