# Supplementary material for: Comparative genome sequencing and analyses of Mycobacterium cosmeticum reveal potential for biodesulfization of gasoline
Source: PLoS One. 2019 Apr 9;14(4):e0214663. doi: 10.1371/journal.pone.0214663 (PMC6456199; doi:10.1371/journal.pone.0214663)
Supplement: S2 Table — (DOCX) [file pone.0214663.s002.docx]

**S2 Table. RAST predicted genes related to gene transfer in the genomes of UM_NYF and UM_RHS.**

|  | **Category** | **Subcategory** | **Subsystem** | **Role** | **Features** |
| --- | --- | --- | --- | --- | --- |
| 1 | RNA Metabolism | RNA processing and modification | RNA pseudouridine syntheses | Ribosomal large subunit pseudouridine synthase A (EC 4.2.1.70) | fig\|6666666.31769.peg.2241 |
| 2 | RNA Metabolism | RNA processing and modification | RNA pseudouridine syntheses | Ribosomal large subunit pseudouridine synthase D (EC 4.2.1.70) | fig\|6666666.31769.peg.2986 |
| 3 | RNA Metabolism | RNA processing and modification | RNA pseudouridine syntheses | Ribosomal large subunit pseudouridine synthase B (EC 4.2.1.70) | fig\|6666666.31769.peg.488 |
| 4 | RNA Metabolism | RNA processing and modification | RNA pseudouridine syntheses | tRNA pseudouridine synthase B (EC 4.2.1.70) | fig\|6666666.31769.peg.1749 |
| 5 | RNA Metabolism | RNA processing and modification | RNA pseudouridine syntheses | tRNA pseudouridine synthase A (EC 4.2.1.70) | fig\|6666666.31769.peg.5166 |
| 6 | RNA Metabolism | RNA processing and modification | tRNA nucleotidyltransferase | tRNA nucleotidyltransferase (EC 2.7.7.21) (EC 2.7.7.25) | fig\|6666666.31769.peg.2573 |
| 7 | RNA Metabolism | RNA processing and modification | Methylthiotransferases | tRNA-i(6)A37 methylthiotransferase | fig\|6666666.31769.peg.1811 |
| 8 | RNA Metabolism | RNA processing and modification | Ribonucleases in Bacillus | Ribonuclease HII (EC 3.1.26.4) | fig\|6666666.31769.peg.1013 |
| 9 | RNA Metabolism | RNA processing and modification | Ribonucleases in Bacillus | Ribonuclease J2 (endoribonuclease in RNA processing) | fig\|6666666.31769.peg.1782 |
| 10 | RNA Metabolism | RNA processing and modification | RNA 3'-terminal phosphate cyclase | RNA 3'-terminal phosphate cyclase (EC 6.5.1.4) | fig\|6666666.31769.peg.2423 |
| 11 | RNA Metabolism | RNA processing and modification | RNA processing and degradation, bacterial | FIG146085: 3'-to-5' oligoribonuclease A, Bacillus type | fig\|6666666.31769.peg.1728 |
| 12 | RNA Metabolism | RNA processing and modification | RNA processing and degradation, bacterial | Ribonuclease III (EC 3.1.26.3) | fig\|6666666.31769.peg.983 |
| 13 | RNA Metabolism | RNA processing and modification | RNA processing and degradation, bacterial | Ribonuclease E inhibitor RraA | fig\|6666666.31769.peg.1293 |
| 14 | RNA Metabolism | RNA processing and modification | RNA processing and degradation, bacterial | Ribonuclease E (EC 3.1.26.12) | fig\|6666666.31769.peg.2653 |
| 15 | RNA Metabolism | RNA processing and modification | RNA processing and degradation, bacterial | 3'-to-5' oligoribonuclease (orn) | fig\|6666666.31769.peg.1995 |
| 16 | RNA Metabolism | RNA processing and modification | RNA methylation | Ribosomal RNA small subunit methyltransferase E (EC 2.1.1.-) | fig\|6666666.31769.peg.5408 |
| 17 | RNA Metabolism | RNA processing and modification | RNA methylation | 23S rRNA (Uracil-5-) -methyltransferase RumA (EC 2.1.1.-) | fig\|6666666.31769.peg.6385 |
| 18 | RNA Metabolism | RNA processing and modification | RNA methylation | SSU rRNA (adenine(1518)-N(6)/adenine(1519)-N(6))-dimethyltransferase (EC 2.1.1.182) | fig\|6666666.31769.peg.3051 |
| 19 | RNA Metabolism | RNA processing and modification | RNA methylation | 23S rRNA (guanosine-2'-O-) -methyltransferase rlmB (EC 2.1.1.-) | fig\|6666666.31769.peg.3771 |
| 20 | RNA Metabolism | RNA processing and modification | RNA methylation | tRNA (guanine46-N7-)-methyltransferase (EC 2.1.1.33) | fig\|6666666.31769.peg.246 |
| 21 | RNA Metabolism | RNA processing and modification | RNA methylation | Ribosomal RNA large subunit methyltransferase N (EC 2.1.1.-) | fig\|6666666.31769.peg.6116 |
| 22 | RNA Metabolism | RNA processing and modification | RNA methylation | tRNA (Guanine37-N1) -methyltransferase (EC 2.1.1.31) | fig\|6666666.31769.peg.1009 |
| 23 | RNA Metabolism | RNA processing and modification | RNA methylation | rRNA small subunit 7-methylguanosine (m7G) methyltransferase GidB | fig\|6666666.31769.peg.2561 |
| 24 | RNA Metabolism | RNA processing and modification | RNA methylation | tRNA (cytidine(34)-2'-O)-methyltransferase (EC 2.1.1.207) | fig\|6666666.31769.peg.1089 |
| 25 | RNA Metabolism | RNA processing and modification | RNA methylation | tRNA-specific 2-thiouridylase MnmA | fig\|6666666.31769.peg.4958 |
| 26 | RNA Metabolism | RNA processing and modification | RNA methylation | 16S rRNA (guanine(966)-N(2))-methyltransferase (EC 2.1.1.171) | fig\|6666666.31769.peg.421 |
| 27 | RNA Metabolism | RNA processing and modification | ATP-dependent RNA helicases, bacterial | Cold-shock DEAD-box protein A | fig\|6666666.31769.peg.5964 |
| **28** | **RNA Metabolism** | **RNA processing and modification** | **16S rRNA modification within P site of ribosome** | **Cell division protein FtsI [Peptidoglycan synthetase] (EC 2.4.1.129)** | **fig\|6666666.31769.peg.1459** |
| **29** | **RNA Metabolism** | **RNA processing and modification** | **16S rRNA modification within P site of ribosome** | **Cell division protein FtsI [Peptidoglycan synthetase] (EC 2.4.1.129)** | **fig\|6666666.31769.peg.3389** |
| **30** | **RNA Metabolism** | **RNA processing and modification** | **16S rRNA modification within P site of ribosome** | **Cell division protein FtsI [Peptidoglycan synthetase] (EC 2.4.1.129)** | **fig\|6666666.31769.peg.6085** |
| **31** | **RNA Metabolism** | **RNA processing and modification** | **16S rRNA modification within P site of ribosome** | **Cell division protein MraZ** | **fig\|6666666.31769.peg.2823** |
| **32** | **RNA Metabolism** | **RNA processing and modification** | **16S rRNA modification within P site of ribosome** | **rRNA small subunit methyltransferase H** | **fig\|6666666.31769.peg.2822** |
| **33** | **RNA Metabolism** | **RNA processing and modification** | **16S rRNA modification within P site of ribosome** | **rRNA small subunit methyltransferase I** | **fig\|6666666.31769.peg.2450** |
| **34** | **RNA Metabolism** | **RNA processing and modification** | **tRNA modification Bacteria** | **FIG137478: Hypothetical protein** | **fig\|6666666.31769.peg.3455** |
| **35** | **RNA Metabolism** | **RNA processing and modification** | **tRNA modification Bacteria** | **Ribosomal large subunit pseudouridine synthase A (EC 4.2.1.70)** | **fig\|6666666.31769.peg.2241** |
| **36** | **RNA Metabolism** | **RNA processing and modification** | **tRNA modification Bacteria** | **Iron-sulfur cluster assembly ATPase protein SufC** | **fig\|6666666.31769.peg.1519** |
| **37** | **RNA Metabolism** | **RNA processing and modification** | **tRNA modification Bacteria** | **tRNA(Ile)-lysidine synthetase** | **fig\|6666666.31769.peg.5813** |
| **38** | **RNA Metabolism** | **RNA processing and modification** | **tRNA modification Bacteria** | **tRNA (guanine46-N7-)-methyltransferase (EC 2.1.1.33)** | **fig\|6666666.31769.peg.246** |
| **39** | **RNA Metabolism** | **RNA processing and modification** | **tRNA modification Bacteria** | **tRNA-specific adenosine-34 deaminase (EC 3.5.4.-)** | **fig\|6666666.31769.peg.4759** |
| **40** | **RNA Metabolism** | **RNA processing and modification** | **tRNA modification Bacteria** | **tRNA-specific adenosine-34 deaminase (EC 3.5.4.-)** | **fig\|6666666.31769.peg.5878** |
| **41** | **RNA Metabolism** | **RNA processing and modification** | **tRNA modification Bacteria** | **GTP cyclohydrolase I (EC 3.5.4.16) type 1** | **fig\|6666666.31769.peg.3113** |
| **42** | **RNA Metabolism** | **RNA processing and modification** | **tRNA modification Bacteria** | **Cysteine desulfurase (EC 2.8.1.7)** | **fig\|6666666.31769.peg.2140** |
| **43** | **RNA Metabolism** | **RNA processing and modification** | **tRNA modification Bacteria** | **Cysteine desulfurase (EC 2.8.1.7)** | **fig\|6666666.31769.peg.2406** |
| **44** | **RNA Metabolism** | **RNA processing and modification** | **tRNA modification Bacteria** | **Cysteine desulfurase (EC 2.8.1.7)** | **fig\|6666666.31769.peg.4957** |
| **45** | **RNA Metabolism** | **RNA processing and modification** | **tRNA modification Bacteria** | **FIG004453: protein YceG like** | **fig\|6666666.31769.peg.4464** |
| **46** | **RNA Metabolism** | **RNA processing and modification** | **tRNA modification Bacteria** | **tRNA-guanine transglycosylase (EC 2.4.2.29)** | **fig\|6666666.31769.peg.5888** |
| **47** | **RNA Metabolism** | **RNA processing and modification** | **tRNA modification Bacteria** | **tRNA-i(6)A37 methylthiotransferase** | **fig\|6666666.31769.peg.1811** |
| **48** | **RNA Metabolism** | **RNA processing and modification** | **tRNA modification Bacteria** | **Ferredoxin, 2Fe-2S** | **fig\|6666666.31769.peg.5769** |
| **49** | **RNA Metabolism** | **RNA processing and modification** | **tRNA modification Bacteria** | **RNA binding methyltransferase FtsJ like** | **fig\|6666666.31769.peg.3216** |
| **50** | **RNA Metabolism** | **RNA processing and modification** | **tRNA modification Bacteria** | **tRNA pseudouridine synthase B (EC 4.2.1.70)** | **fig\|6666666.31769.peg.1749** |
| **51** | **RNA Metabolism** | **RNA processing and modification** | **tRNA modification Bacteria** | **Iron-sulfur cluster assembly protein SufD** | **fig\|6666666.31769.peg.1520** |
| **52** | **RNA Metabolism** | **RNA processing and modification** | **tRNA modification Bacteria** | **tRNA (Guanine37-N1) -methyltransferase (EC 2.1.1.31)** | **fig\|6666666.31769.peg.1009** |
| **53** | **RNA Metabolism** | **RNA processing and modification** | **tRNA modification Bacteria** | **tRNA dihydrouridine synthase B (EC 1.-.-.-)** | **fig\|6666666.31769.peg.5113** |
| **54** | **RNA Metabolism** | **RNA processing and modification** | **tRNA modification Bacteria** | **Cytidine deaminase (EC 3.5.4.5)** | **fig\|6666666.31769.peg.2170** |
| **55** | **RNA Metabolism** | **RNA processing and modification** | **tRNA modification Bacteria** | **tRNA dimethylallyltransferase (EC 2.5.1.75)** | **fig\|6666666.31769.peg.6432** |
| **56** | **RNA Metabolism** | **RNA processing and modification** | **tRNA modification Bacteria** | **Cysteine desulfurase (EC 2.8.1.7), SufS subfamily** | **fig\|6666666.31769.peg.1518** |
| **57** | **RNA Metabolism** | **RNA processing and modification** | **tRNA modification Bacteria** | **Iron-sulfur cluster assembly protein SufB** | **fig\|6666666.31769.peg.1521** |
| **58** | **RNA Metabolism** | **RNA processing and modification** | **tRNA modification Bacteria** | **glutamyl-Q-tRNA synthetase** | **fig\|6666666.31769.peg.5894** |
| **59** | **RNA Metabolism** | **RNA processing and modification** | **tRNA modification Bacteria** | **tRNA pseudouridine synthase A (EC 4.2.1.70)** | **fig\|6666666.31769.peg.5166** |
| 60 | RNA Metabolism | RNA processing and modification | Ribonuclease H | Ribonuclease HI (EC 3.1.26.4) | fig\|6666666.31769.peg.6007 |
| 61 | RNA Metabolism | RNA processing and modification | Ribonuclease H | Ribonuclease HII (EC 3.1.26.4) | fig\|6666666.31769.peg.1013 |
| 62 | RNA Metabolism | RNA processing and modification | Ribonuclease H | Protein often found in Actinomycetes clustered with signal peptidase and/or RNaseHII | fig\|6666666.31769.peg.1014 |
| 63 | RNA Metabolism | RNA processing and modification | Queuosine-Archaeosine Biosynthesis | Peptidyl-prolyl cis-trans isomerase (EC 5.2.1.8) | fig\|6666666.31769.peg.1452 |
| 64 | RNA Metabolism | RNA processing and modification | Queuosine-Archaeosine Biosynthesis | Peptidyl-prolyl cis-trans isomerase (EC 5.2.1.8) | fig\|6666666.31769.peg.4504 |
| 65 | RNA Metabolism | RNA processing and modification | Queuosine-Archaeosine Biosynthesis | Peptidyl-prolyl cis-trans isomerase (EC 5.2.1.8) | fig\|6666666.31769.peg.4505 |
| 66 | RNA Metabolism | RNA processing and modification | Queuosine-Archaeosine Biosynthesis | GTP cyclohydrolase I (EC 3.5.4.16) type 1 | fig\|6666666.31769.peg.3113 |
| 67 | RNA Metabolism | RNA processing and modification | Queuosine-Archaeosine Biosynthesis | Putative preQ0 transporter | fig\|6666666.31769.peg.754 |
| 68 | RNA Metabolism | RNA processing and modification | Queuosine-Archaeosine Biosynthesis | Permease of the drug/metabolite transporter (DMT) superfamily | fig\|6666666.31769.peg.2072 |
| 69 | RNA Metabolism | RNA processing and modification | Queuosine-Archaeosine Biosynthesis | Permease of the drug/metabolite transporter (DMT) superfamily | fig\|6666666.31769.peg.3314 |
| 70 | RNA Metabolism | RNA processing and modification | Queuosine-Archaeosine Biosynthesis | glutamyl-Q-tRNA synthetase | fig\|6666666.31769.peg.5894 |
| 71 | RNA Metabolism | RNA processing and modification | Queuosine-Archaeosine Biosynthesis | tRNA-guanine transglycosylase (EC 2.4.2.29) | fig\|6666666.31769.peg.5888 |
| 72 | RNA Metabolism | RNA processing and modification | tRNA processing | Ribonuclease P protein component (EC 3.1.26.5) | fig\|6666666.31769.peg.2557 |
| 73 | RNA Metabolism | RNA processing and modification | tRNA processing | tRNA-i(6)A37 methylthiotransferase | fig\|6666666.31769.peg.1811 |
| 74 | RNA Metabolism | RNA processing and modification | tRNA processing | Ribonuclease D (EC 3.1.26.3) | fig\|6666666.31769.peg.6381 |
| 75 | RNA Metabolism | RNA processing and modification | tRNA processing | tRNA pseudouridine synthase B (EC 4.2.1.70) | fig\|6666666.31769.peg.1749 |
| 76 | RNA Metabolism | RNA processing and modification | tRNA processing | tRNA(Ile)-lysidine synthetase | fig\|6666666.31769.peg.5813 |
| 77 | RNA Metabolism | RNA processing and modification | tRNA processing | tRNA-specific adenosine-34 deaminase (EC 3.5.4.-) | fig\|6666666.31769.peg.4759 |
| 78 | RNA Metabolism | RNA processing and modification | tRNA processing | tRNA-specific adenosine-34 deaminase (EC 3.5.4.-) | fig\|6666666.31769.peg.5878 |
| 79 | RNA Metabolism | RNA processing and modification | tRNA processing | tRNA dimethylallyltransferase (EC 2.5.1.75) | fig\|6666666.31769.peg.6432 |
| 80 | RNA Metabolism | RNA processing and modification | tRNA processing | Ribonuclease PH (EC 2.7.7.56) | fig\|6666666.31769.peg.583 |
| 81 | RNA Metabolism | RNA processing and modification | tRNA processing | tRNA pseudouridine synthase A (EC 4.2.1.70) | fig\|6666666.31769.peg.5166 |
| 82 | RNA Metabolism | Transcription | Transcription initiation, bacterial sigma factors | RNA polymerase sigma-54 factor RpoN | fig\|6666666.31769.peg.5470 |
| 83 | RNA Metabolism | Transcription | Transcription initiation, bacterial sigma factors | RNA polymerase sigma-54 factor RpoN | fig\|6666666.31769.peg.6578 |
| 84 | RNA Metabolism | Transcription | Transcription initiation, bacterial sigma factors | RNA polymerase sigma factor RpoD | fig\|6666666.31769.peg.6399 |
| 85 | RNA Metabolism | Transcription | Transcription initiation, bacterial sigma factors | RNA polymerase sigma-70 factor | fig\|6666666.31769.peg.1906 |
| 86 | RNA Metabolism | Transcription | Transcription initiation, bacterial sigma factors | RNA polymerase sigma-70 factor | fig\|6666666.31769.peg.3491 |
| 87 | RNA Metabolism | Transcription | Transcription initiation, bacterial sigma factors | RNA polymerase sigma-70 factor | fig\|6666666.31769.peg.3844 |
| 88 | RNA Metabolism | Transcription | Transcription initiation, bacterial sigma factors | RNA polymerase sigma-70 factor | fig\|6666666.31769.peg.6037 |
| 89 | RNA Metabolism | Transcription | Transcription initiation, bacterial sigma factors | RNA polymerase sigma-70 factor | fig\|6666666.31769.peg.6093 |
| 90 | RNA Metabolism | Transcription | Transcription initiation, bacterial sigma factors | RNA polymerase sigma factor RpoE | fig\|6666666.31769.peg.1236 |
| 91 | RNA Metabolism | Transcription | Transcription initiation, bacterial sigma factors | RNA polymerase sigma factor SigB | fig\|6666666.31769.peg.858 |
| 92 | RNA Metabolism | Transcription | Transcription initiation, bacterial sigma factors | RNA polymerase sigma factor SigB | fig\|6666666.31769.peg.6411 |
| 93 | RNA Metabolism | Transcription | RNA polymerase bacterial | DNA-directed RNA polymerase alpha subunit (EC 2.7.7.6) | fig\|6666666.31769.peg.5168 |
| 94 | RNA Metabolism | Transcription | RNA polymerase bacterial | DNA-directed RNA polymerase beta' subunit (EC 2.7.7.6) | fig\|6666666.31769.peg.1813 |
| 95 | RNA Metabolism | Transcription | RNA polymerase bacterial | DNA-directed RNA polymerase omega subunit (EC 2.7.7.6) | fig\|6666666.31769.peg.4431 |
| 96 | RNA Metabolism | Transcription | RNA polymerase bacterial | DNA-directed RNA polymerase beta subunit (EC 2.7.7.6) | fig\|6666666.31769.peg.1814 |
| 97 | RNA Metabolism | Transcription | Transcription factors bacterial | Transcription termination protein NusB | fig\|6666666.31769.peg.4454 |
| 98 | RNA Metabolism | Transcription | Transcription factors bacterial | Transcription accessory protein (S1 RNA-binding domain) | fig\|6666666.31769.peg.469 |
| 99 | RNA Metabolism | Transcription | Transcription factors bacterial | Transcription elongation factor GreA | fig\|6666666.31769.peg.2359 |
| 100 | RNA Metabolism | Transcription | Transcription factors bacterial | Transcription termination factor Rho | fig\|6666666.31769.peg.6516 |
| 101 | RNA Metabolism | Transcription | Transcription factors bacterial | Transcription antitermination protein NusG | fig\|6666666.31769.peg.2114 |
| 102 | RNA Metabolism | Transcription | Transcription factors bacterial | Transcription-repair coupling factor | fig\|6666666.31769.peg.3073 |
| 103 | RNA Metabolism | Transcription | Transcription factors bacterial | FIG000325: clustered with transcription termination protein NusA | fig\|6666666.31769.peg.2584 |
| 104 | RNA Metabolism | Transcription | Transcription factors bacterial | Transcription termination protein NusA | fig\|6666666.31769.peg.2585 |
| 105 | RNA Metabolism | Transcription | Rrf2 family transcriptional regulators | Predicted transcriptional regulator of sulfate adenylyltransferase, Rrf2 family | fig\|6666666.31769.peg.6535 |
| 106 | RNA Metabolism | Transcription | Rrf2 family transcriptional regulators | Nitrite-sensitive transcriptional repressor NsrR | fig\|6666666.31769.peg.2121 |
| 107 | RNA Metabolism | Transcription | Rrf2 family transcriptional regulators | Nitrite-sensitive transcriptional repressor NsrR | fig\|6666666.31769.peg.4134 |
