# Supplementary material for: Comparative genome sequencing and analyses of Mycobacterium cosmeticum reveal potential for biodesulfization of gasoline
Source: PLoS One. 2019 Apr 9;14(4):e0214663. doi: 10.1371/journal.pone.0214663 (PMC6456199; doi:10.1371/journal.pone.0214663)
Supplement: S3 Table — (DOCX) [file pone.0214663.s003.docx]

**S3 Table. Genomic Islands present in UM_RHS and UM_NYF**

| **GI** | **GI length** | **GC content** | **UM_RHS** | **UM_NYF** |
| --- | --- | --- | --- | --- |
| GI1 | 15,225 | 63.9 | / | / |
| GI2 | 4,724 | 61.8 | / | X |
| GI3 | 9,516 | 61.5 | / | / |
| GI4 | 7,084 | 63.6 | / | / |
| GI5 | 19,034 | 62.1 | / | / |
| GI6 | 5,994 | 65 | / | / |
| GI7 | 7,765 | 63.6 | / | / |
| GI8 | 5,506 | 67.05 | / | / |
| GI9 | 4,627 | 66.1 | / | X |
| GI10 | 4,785 | 62.3 | / | X |
| GI11 | 10,210 | 64.4 | / | / |
| GI12 | 9,030 | 62.3 | / | / |
| GI13 | 14,737 | 61.5 | / | / |
| GI14 | 6,660 | 64.2 | / | / |
| GI15 | 5,642 | 64.1 | / | / |
| GI16 | 12,462 | 62.9 | / | / |
| GI17 | 6,081 | 60.3 | / | / |
| GI18 | 5,090 | 63.6 | / | / |
| GI19 | 28,671 | 62.7 | / | / |
| GI20 | 5,231 | 64.9 | / | / |
| GI21 | 6,649 | 68.1 | / | / |
| GI22 | 6,159 | 63.1 | / | / |
| GI23 | 5,160 | 62.8 | / | / |
| GI24 | 5,884 | 61.5 | / | / |
| GI25 | 15,528 | 62.8 | / | / |
| GI26 | 4,163 | 65.8 | / | / |
| GI27 | 4,667 | 66 | / | / |
| GI28 | 4,817 | 65 | / | / |
| GI29 | 4,511 | 65.5 | / | / |
| GI30 | 5,971 | 61.4 | / | / |
| GI31 | 10,534 | 62.8 | / | / |
| GI32 | 5,966 | 66 | / | / |
| GI33 | 10,260 | 62.5 | / | / |
| GI34 | 7,775 | 62.8 | / | X |
| GI35 | 4,948 | 61.8 | / | / |
| GI36 | 4,101 | 62.3 | / | / |
| GI37 | 7,332 | 62.2 | / | / |
| GI38 | 9,929 | 61.3 | / | X |
| GI39 | 9,790 | 62.2 | / | / |
| GI40 | 4,442 | 62.5 | / | / |
| GI41 | 32,656 | 61.3 | X | / |
| GI42 | 6,684 | 64.4 | X | / |

‘/’ indicates present; ‘X’ indicates absent
