# Supplementary material for: Comparative genome sequencing and analyses of Mycobacterium cosmeticum reveal potential for biodesulfization of gasoline
Source: PLoS One. 2019 Apr 9;14(4):e0214663. doi: 10.1371/journal.pone.0214663 (PMC6456199; doi:10.1371/journal.pone.0214663)
Supplement: S1 Fig — (PDF) [file pone.0214663.s004.pdf]

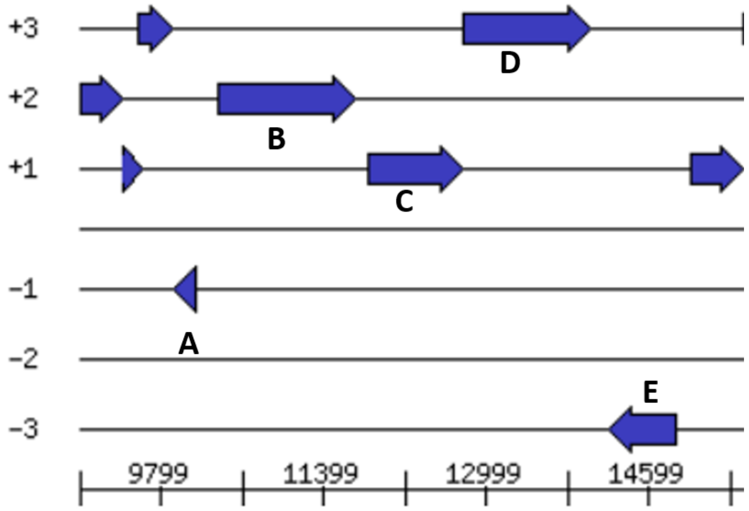

S1 Fig. The graphical representation of GI35. GI35 is located in the contig87 from 9,931 to 14,878. GI35 consists of 5 genes: A – Transcriptional regulator, IclR family; B - Dibenzothiophene desulfurization enzyme A; C - Dibenzothiophene desulfurization enzyme B ; D - Dibenzothiophene desulfurization enzyme C; E - Transcriptional regulator, TetR family (+3 to -3 indicated the open reading frame).
